# Early changes in ambulatory electrocardiography after transcatheter closure in patients with atrial septal defect and factors affecting heart rate variability

**DOI:** 10.1186/s12872-020-01699-4

**Published:** 2020-09-11

**Authors:** Zhenyang Su, Qing Cao, Hao Zhang, Wei Sun, Haifeng Zhang, Yanhui Sheng, Rong Yang, Xiangqing Kong

**Affiliations:** Department of Cardiology, The First Affiliated Hospital of Nanjing Medical University, No. 300, Guangzhou Road, Nanjing, 210029 China

**Keywords:** Atrial septal defect, Ambulatory electrocardiography, Heart rate variability, Transcatheter closure

## Abstract

**Background:**

Factors affecting heart rate variability (HRV) in patients with atrial septal defect (ASD) have not been clarified. This study sought to identify those factors and establish a preliminary risk model.

**Methods:**

A total of 154 patients with ASD who underwent transcatheter closure and met the study requirements were analyzed in this study. Moreover, 26 patients with patent foramen ovale (PFO) were enrolled in our study as a control group. All patients underwent echocardiography and ambulatory electrocardiography before and one day after the procedure.

**Results:**

The standard deviation of all normal-to-normal (NN) intervals (SDNN) and the standard deviation of the averages of the NN intervals in all 5 min segments of the entire recording (SDANN) were significantly higher and the heart rate was lower after closure than before closure in patients with ASD (SDNN: 6.08, 95% CI 3.00 to 9.15, *p* < 0.001; SDANN: 7.57, 95% CI 4.50 to 10.64, *p <* 0.001; heart rate: -1.17, 95% CI − 2.86 to − 0.48, *p* = 0.006). Multiple regression analyses indicated that age, sex, defect diameter, heart rate and diabetes were significantly associated with HRV indices (SDNN: *R*^*2*^ = 0.415; *P <* 0.001). SDNN and SDANN had obvious correlations with right ventricular systolic pressure (SDNN: *R =* − 0.370, *p* < 0.001; SDANN: *R* = − 0.360, *p <* 0.001).

**Conclusions:**

Factors affecting HRV in patients with ASD include age, sex, heart rate, defect size and diabetes. Furthermore, right ventricular systolic pressure plays an important role in the change in HRV.

## Introduction

Atrial septal defect (ASD) is one of the most common types of congenital heart disease. The interatrial defect results in a left-to-right shunt. It is generally accepted that defects smaller than 10 mm in diameter rarely result in changes in the heart structure [1]. When the defect diameter is large enough to cause significant hemodynamic changes, a longstanding shunt may result in impaired heart structure and cardiac insufficiency. Atrial electrical remodeling resulting from chronic right atrial stretch in ASD patients frequently manifests as conduction delay at the crista terminalis and impaired sinus node function [2]. A recent study suggested that later arrhythmia can be predicted by measurement of left atrial volume [3].

Heart rate variability (HRV), which is known as a noninvasive, reliable indication of autonomic nervous system activity, has a unique manifestation in patients with ASD. It has been shown that HRV is reduced in children with ASD [4, 5]. Surgery or transcatheter closure can improve HRV in pediatric patients [6–8]. However, there is not much research on HRV in adult patients with ASD. The factors that affect HRV in patients with ASD are not yet clear.

The aim of the present study was to evaluate the changes in HRV and heart rate in patients with ASD before and after transcatheter closure and explore factors affecting HRV indices in these patients.

## Methods

### Study population and protocol

The study population consisted of patients who underwent transcatheter closure of ASDs at the First Affiliated Hospital of Nanjing Medical University between October 2017 and December 2019. The exclusion criteria for this study were as follows: (1) concurrent presence of other congenital heart structural abnormalities, (2) diagnosis of serious atrial arrhythmias such as atrial fibrillation, atrial flutter and persistent atrial tachycardia before closure, (3) a history of acute myocardial infarction, or (4) receiving medications such as beta-blockers that could influence HRV measurements. During the same study period, patients with patent foramen ovale (PFO) device closure were enrolled in the study as the interventional control group. All patients underwent echocardiography and twenty-four-hour ambulatory electrocardiography before and 1 day after the procedure. All measurement data was recorded by two different blinded operators. The study protocol was approved by the Ethics Committee of the First Affiliated Hospital of Nanjing Medical University.

### Procedure of transcatheter closure

The two groups of patients underwent an intervention with the same method: puncture the femoral vein, establishment of a delivery system and insertion of the device into the defect/oval foramen. Real-time two-dimensional transthoracic echocardiography was used for guidance during the process. Hemodynamic parameters were measured by a right heart catheter before closure in patients with ASD. Antiplatelet drugs were used in all patients after device implantation.

### Ambulatory electrocardiographic recordings and HRV indices

Ambulatory electrocardiographic recordings were performed using an ambulatory 12-channel electrocardiogram (ECG) recorder. The recording time of all patients was not less than 23 h. Mean heart rate and HRV indices were included in the study. The main indices of HRV included time-domain and frequency-domain indices. It has been confirmed that there is a strong correlation between time-domain and frequency-domain indices, and time-domain indices are more valuable and easy to analyze in the 24-h record [9–11]. Therefore, time-domain indices, which include the standard deviation of all normal-to-normal (NN) intervals (SDNN), the standard deviation of the averages of the NN intervals in all 5 min segments of the entire recording (SDANN), the square root of the mean of the sum of the squares of differences between adjacent NN intervals (RMSSD) and the percentage of adjacent NN intervals that differ from each other by more than 50 ms (pNN50), are the main research objects of HRV in our study. In particular, SDNN is taken as a dependable standard for medical stratification of cardiac risk when recorded over twenty-four hours [10, 12].

### Statistical analysis

Continuous variables are expressed as the mean ± standard deviation. Categorical variables are expressed as frequencies and percentages. All continuous variables obey normal distribution according to Kolmogorov-Smirnov test and Q-Q plot. Two-tailed two-sample t-test and paired t-test were used respectively to compare continuous variables between and within groups. Bonferroni correction was used to correct the significance level. The chi-square test was used to compare groups of categorical variables. Correlations were analyzed by using Pearson’s correlation coefficient. Multiple linear regression analyses were performed to estimate factors influencing the HRV indices. Statistical significance was set at *p* < 0.05. All statistical analyses were carried out using SPSS version 25.0 for Windows (SPSS Inc., Chicago, IL, USA). Statistical graphics were drawn with GraphPad Prism 7.0 for Windows (GraphPad Software Inc., San Diego, CA, USA).

## Results

### Baseline characteristics

The clinical characteristics of the enrolled ASD patients and PFO patients are presented in Table 1. No significant differences in age, sex ratio, body weight, blood pressure, hypertension or diabetes mellitus were found between the two groups. Defect diameters in patients with ASD were measured through transesophageal echocardiogram before closure.

**Table 1:** Baseline Characteristics of the two groups of patients

Factor	Group ASD (***n*** = 154)	Group PFO (***n*** = 26)	***p*** Value
Age, years	39.54 ± 15.62	40.58 ± 14.22	0.750
Male sex, n (%)	43 (27.9)	5 (19.2)	0.357
Weight, kg	60.65 ± 10.49	60.59 ± 11.09	0.979
Systolic BP, mmHg	119.59 ± 12.76	120.23 ± 13.71	0.815
Diastolic BP, mmHg	75.74 ± 9.23	79.08 ± 8.55	0.087
Hypertension, n (%)	34 (22.1)	3 (11.5)	0.297
Diabetes, n (%)	6 (3.9)	1 (3.8)	1.000

### Changes in ambulatory electrocardiography and transthoracic echocardiography parameters after transcatheter closure

The data for ambulatory electrocardiography and transthoracic echocardiography before and after interventional therapy are summarized in Table 2. There were significant differences between the two groups in each HRV index before transcatheter closure. In the ASD group, the SDNN and SDANN were significantly higher and the mean heart rate was lower after closure than before closure (SDNN: 6.08, 95% CI 3.00 to 9.15, *p* < 0.001; SDANN: 7.57, 95% CI 4.50 to 10.64, *p <* 0.001; heart rate: -1.17, 95% CI − 2.86 to − 0.48, *p* = 0.006). In the PFO group, all HRV indices, including SDNN, SDANN, RMSSD and PNN50, were significantly lower after closure than before closure, and the mean heart rate increased after closure (SDNN: -29.62, 95% CI − 42.05 to − 17.18, *p* < 0.001; SDANN: -22.89, 95% CI − 33.61 to − 12.16, *p* < 0.001; RMSSD: -10.46, 95% CI − 33.61 to − 12.16, *p <* 0.001; PNN50: -7.25, 95% CI − 10.24 to − 4.26, *p <* 0.001; mean heart rate: 5.81, 95% CI 2.70 to 8.92, *p* = 0.001). In addition, there were immediate, significant changes in heart structure after transcatheter closure in patients with ASD. Right atrial diameter (RAD) and right ventricle diameter (RVD) were significantly lower than before (RAD: -3.33, 95% CI − 4.08 to − 2.57, *p* < 0.001; RVD: -3.20, 95% CI − 3.88 to − 2.53, *p <* 0.001). Although the difference was small, left ventricular end diastolic diameter (LVEDD) increased significantly after transcatheter closure (LVEDD: 0.82, 95% CI 0.32 to 1.31, *p* = 0.001). These changes were not significant in patients with PFO.

**Table 2:** The ambulatory electrocardiography and transthoracic echocardiography data before and after closure

	Group ASD (***n*** = 154)			Group PFO (***n*** = 26)			P0^*^
	Before	After	***p*** Value	Before	After	***p*** Value	
**HRV indices and HR**							
SDNN, ms	98.70 ± 24.08	104.78 ± 25.67	< 0.001	134.08 ± 33.80	104.46 ± 22.64	< 0.001	< 0.001
SDANN, ms	84.85 ± 22.04	92.42 ± 25.11	< 0.001	115.62 ± 31.87	92.73 ± 22.57	< 0.001	< 0.001
RMSSD, ms	27.83 ± 10.81	26.21 ± 10.73	0.082	34.23 ± 11.80	23.77 ± 10.97	< 0.001	0.006
PNN50, %	8.08 ± 7.74	7.33 ± 7.35	0.262	13.10 ± 9.89	5.84 ± 7.27	< 0.001	0.004
HR, bpm	76.81 ± 9.10	75.14 ± 9.06	0.006	75.58 ± 10.78	81.38 ± 8.17	0.001	0.537
**TTE parameters**							
LAD, mm	32.43 ± 4.32	31.92 ± 4.54	0.088	31.00 ± 3.49	29.58 ± 4.49	0.054	0.124
LVESD, mm	27.95 ± 2.98	28.20 ± 2.72	0.199	29.54 ± 2.21	28.63 ± 2.50	0.055	0.013
LVEDD, mm	42.81 ± 4.03	43.63 ± 3.66	0.001	45.38 ± 3.70	44.13 ± 3.63	0.042	0.004
RAD, mm	38.13 ± 5.71	34.80 ± 4.58	< 0.001	29.75 ± 3.01	28.88 ± 3.22	0.131	< 0.001
RVD, mm	38.92 ± 5.01	35.72 ± 4.70	< 0.001	29.17 ± 3.32	29.21 ± 3.59	0.957	< 0.001
LVEF, %	64.12 ± 4.06	65.00 ± 2.92	0.019	64.15 ± 2.00	64.53 ± 4.04	0.697	0.971

^*^P0: *p*-values between the values prior to the procedure in the patient and control groups; *P* value threshold of 0.017 was used after Bonferroni correction (0.05*(1/3));

*P* value threshold of 0.017 was used after Bonferroni correction (0.05*(1/3)); *SDNN* the standard deviation of all normal-to-normal (NN) intervals, *SDANN* the standard deviation of the averages of NN intervals in all 5 min segments of the entire recording, *RMSSD* the square root of the mean of the sum of the squares of differences between adjacent NN intervals, *pNN50* the percentage of adjacent NN intervals that differ from each other by more than 50 msm, *HR* heart rate, *LAD* left atrium diameter, *LVESD* left ventricular end systolic diameter, *LVEDD* left ventricular end diastolic diameter, *RAD* right atrium diameter, *RVD* right ventricular diameter, *LVEF* left ventricular ejection fraction

**Fig. 1:**
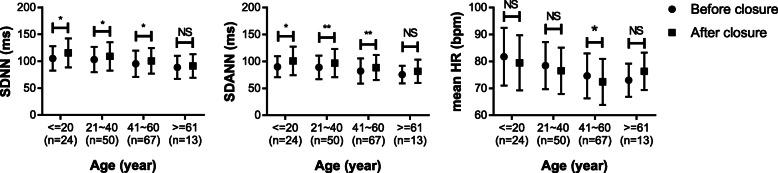
Changes in SDNN, SDANN and mean heart rate before and after closure at different ages. Significance levels: NS, no significance; *P < 0.05, **P < 0.01, ***P < 0.001

Patients with ASD were divided according to age stratification: ≤20 years old, 21 ~ 40 years old, 41 ~ 60 years old, ≥60 years old (Fig. 1). At the age of 20 years or younger, 21 ~ 40 years and 41 ~ 60 years, SDNN and SDANN increased significantly after closure. At the age of 41 ~ 60 years, the mean heart rate reduced significantly after closure. Our data showed a trend of increased heart rate after closure at the age of 61 years or older, but the change did not reach statistical significance.

### Factors affecting HRV in patients with ASD before closure

**Fig. 2:**
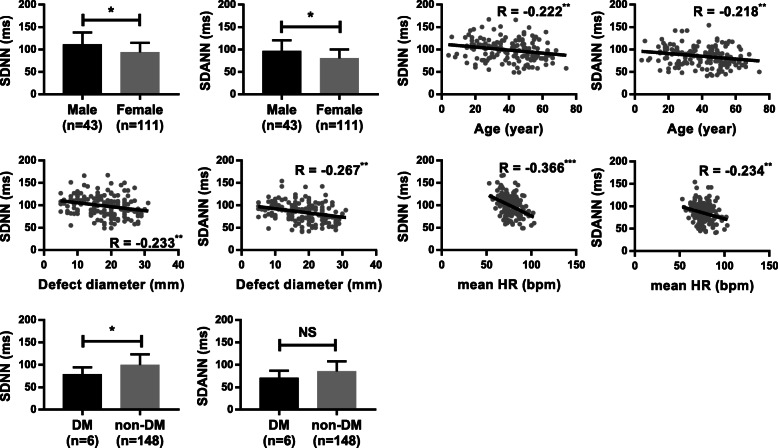
Factors affecting HRV in patients with ASD. Comparison of SDNN and SDANN between different sexes. Linear regression and Pearson’s correlation analyses showing the relationship of age, defect diameter and mean heart rate with SDNN and SDANN. Comparison of SDNN and SDANN between diabetic and nondiabetic patients. R, Pearson’s correlation coefficient. Significance levels: NS, no significance; *p < 0.05, **p < 0.01, ***p < 0.001

To further understand factors affecting HRV in patients with ASD, linear regression and correlation analyses were performed (Fig. 2). SDNN and SDANN were the main research indicators of HRV. The impact of sex on HRV was compared. The values of SDNN and SDANN were significantly higher in male patients than in female patients. Linear regression and correlation analyses demonstrated that age, defect diameter and mean heart rate were significantly inversely correlated with SDNN and SDANN. Additionally, diabetic patients had significantly lower SDNN values ​​than nondiabetic patients. No significant differences in SDANN were found between diabetic and nondiabetic patients. Thus, multiple regression analyses indicated that female sex, old age, large defect diameter, fast heart rate and diabetes mellitus were predictors of HRV reduction. The regression equation was as follows (Table 3): SDNN (ms) = 258.94–11.42 × [Sex] - 0.49 × [Age] - 1.09 × [Defect diameter] -1.32 × [Mean heart rate] - 18.99 × [Diabetes]. The R^2^ (0.415; *P* < 0.001) value indicated that 41.5% of the variance in SDNN could be estimated using this model. In this equation, male sex is assigned a value of one, and female sex is assigned a value of two. The presence of diabetes is assigned one, and absence of diabetes is assigned zero.

**Table 3:** Multiple linear regression analyses on SDNN and SDANN

Dependent variable	Variable	Unstandardized coefficient		Standardized β	***p*** Value	R^**2**^
		β	Std. Error			
SDNN	Age	−0.487	0.107	− 0.316	< 0.001	0.415
	Sex	−11.416	3.451	−0.213	0.001	
	Defect diameter	−1.091	0.245	− 0.287	< 0.001	
	Heart rate	−1.316	0.181	−0.498	< 0.001	
	Diabetes	−18.990	8.059	−0.153	0.02	
SDANN	Age	−0.403	0.105	−0.285	< 0.001	0.296
	Sex	−11.732	3.449	−0.240	0.001	
	Defect diameter	−0.969	0.242	−0.279	< 0.001	
	Heart rate	−0.840	0.181	−0.347	< 0.001	

*SDNN* the standard deviation of all normal-to-normal (NN) intervals, *SDANN* the standard deviation of the averages of NN intervals in all 5 min segments of the entire recording

**Fig. 3:**
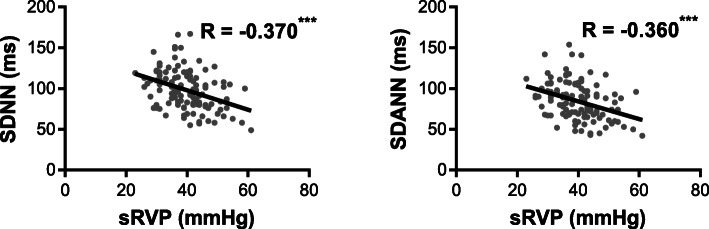
Relationship between right ventricular systolic pressure and HRV. Linear regression and Pearson’s correlation analyses showing the relationship of right ventricular systolic pressure (sRVP) with SDNN and SDANN. R, Pearson’s correlation coefficient. Significance levels: NS, no significance; *p < 0.05, **p < 0.01, ***p < 0.001

Furthermore, after excluding patients younger than 20 years of age or with diabetes, correlations between HRV and pressure in the various chambers of the heart as measured by the right heart catheter were assessed (Fig. 3). SDNN and SDANN have obvious correlations with right ventricular systolic pressure (SDNN: *R* = − 0.370, *p* < 0.001; SDANN: *R* = − 0.360, *p* < 0.001; *n* = 124).

## Discussion

HRV, as a noninvasive examination to reflect the influence of sympathetic and vagal activity on the sinus node, is widely used in the diagnosis and prognostic assessment of clinical diseases. It has been confirmed that HRV is a strong and independent predictor of mortality in acute myocardial infarction [13]. Reduced HRV is also a marker of increased risk for mortality in healthy people without cardiac disease [12, 14]. Changes in HRV in patients with ASD have also been reported.

Several studies have reported the effects of aging and sex on HRV [15–18]. While the linear relationship between HRV and age is controversial, there is no doubt that time-domain measurements of HRV decrease with age. Compared to men, women have lower HRV. In the present study, both SDNN and SDANN in males were significantly lower than those in females among patients with ASD. The values of SDNN and SDANN showed linear downward trends with age. This may be caused by a decrease in autonomic nerve activity with age.

The results of our study showed that SDNN and SDANN decrease as the defect diameter increases. The size of the defect determines the scale of the left-to-right shunt. The overload of volume and pressure on the right heart caused by a longstanding shunt leads to chamber enlargement and elevated pulmonary artery pressure. Chronic right atrial stretch could cause sinus node dysfunction [2]. This may reduce the reactivity of the sinus node to autonomic nerve activity. Persistent pressure overload results in myocardial impairment and pulmonary hypertension [19–21]. It has been reported that patients with pulmonary hypertension have a marked alteration in cardiac autonomic activity [22]. Our study showed that SDNN and SDANN had negative linear correlations with right ventricular systolic pressure and pulmonary diastolic pressure. After transcatheter closure to block the shunt, the HRV increased, and the diameter of the right atrium and ventricle decreased. Changes in the right atrium and ventricular pressure and extension are intermediate factors in the process of HRV change. The control group comprised patients with PFO. Since there was no left-to-right shunt, the above situations were not observed in the control group.

The mean heart rate is a strong clinical determinant of HRV [12, 23]. Oliver et al. [23] noted that there is a universal exponential decay-like relationship between HRV and heart rate. Excessive resting heart rate could increase the risk of all-cause mortality [24]. The results of our study show that there was a negative linear correlation between HRV and mean heart rate in patients with ASD, and improvement in HRV and reduction in heart rate appeared after closure. This may mean that transcatheter closure could reduce the risk of cardiac death in patients with ASD and improve prognosis.

Moreover, patients with diabetes or prediabetes have lower HRV [25, 26]. Diabetes-related cardiac autonomic dysfunction plays an important role in the decrease in HRV [27]. The results of our analyses show that having diabetes is associated with lower HRV, which also occurs in patients with ASD. In addition, our data did not show a linear relationship between blood pressure and HRV in patients with ASD, and there was no significant difference between HRV in hypertensive patients and HRV in non-hypertensive patients.

We included patients with PFO in the control group because both types of patients had congenital disorders of the atrial septum and both required the same procedure for interventional closure. The difference is that patients with ASD have obvious left-to-right shunts and significant changes in cardiac structure and function. There is almost no shunt in patients with PFO, so the postoperative changes are only related to the operation itself. Transcatheter closure could have a temporary negative effect on heart function. The specific manifestations of this effect were shown to be a decrease in HRV and an increase in heart rate after closure. This was a hypothesis and needs further research to confirm. Due to this effect, the immediate changes in HRV and HR of patients with ASD after transcatheter closure would be smaller than expected. This made the changes of HRV indices and HR after closure in patients with ASD statistically significant, but slight from a clinical perspective.

Therefore, we consider that the reduced HRV reflects the degree of heart damage caused by shunt. It is necessary to close the defects in patients with ASD in time, especially those with large defects. For patients who have not yet experienced a significant reduction in HRV, early closure is also beneficial, because age is also one of the factors affecting HRV. We believe that the above conclusions strengthen the indications for transcatheter closure. And postoperative changes in HRV can be used to evaluate the recovery of heart function, which shows the effectiveness and necessity of the percutaneous treatment.

### Study limitations

Several limitations exist in this study. Because of the safety of the operation and the absence of short-term arrhythmias worthy of attention after transcatheter closure, most patients failed to complete follow-up ambulatory electrocardiograms. The follow-up time was not long enough in patients who underwent follow-up. Furthermore, HRV may also increase under pathological conditions, such as some special types of acute ischemic stroke [28]. Additionally, cerebral thromboembolism in patients with congenital heart disease is not uncommon, and these patients need more individualized attention.

Further research can be anticipated to expound the relationship between HRV and cardiac volume and intracavitary pressure. Therefore, HRV could be used to predict the long-term prognosis of patients after interventional closure and provide more sufficient evidence for the choice of closure timing. Whether the long-term changes in postoperative HRV in patients of different ages are significant is also a question worthy of further research.

## Conclusions

Patients with ASD have significantly reduced HRV and increased heart rate, which can be improved by transcatheter closure. There is a negative correlation between HRV and heart rate. Other factors affecting HRV in patients with ASD include age, sex, diabetes, and defect size. Furthermore, right ventricular systolic pressure plays an important role in the change in HRV.

## Data Availability

The datasets used and/or analyzed during the current study are available from the corresponding author on reasonable request.

## Abbreviations

ASD: Atrial septal defect
HRV: Heart rate variability
PFO: Patent foramen ovale
ECG: Electrocardiogram
SDNN: Standard deviation of all normal-to-normal (NN) intervals
SDANN: Standard deviation of the averages of NN intervals in all 5 min segments of the entire recording
RMSSD: Square root of the mean of the sum of the squares of differences between adjacent NN intervals
pNN50: Percentage of adjacent NN intervals that differ from each other by more than 50 ms
HR: Heart rate
LAD: Left atrium diameter
LVESD: Left ventricular end systolic diameter
LVEDD: Left ventricular end diastolic diameter
RAD: Right atrium diameter
RVD: Right ventricular diameter
LVEF: Left ventricular ejection fraction
